# Opportunities and challenges for hybrid immunoaffinity LC–MS approach for quantitative analysis of protein biomarkers

**DOI:** 10.4155/fsoa-2017-0148

**Published:** 2018-02-14

**Authors:** Yue Zhao, Huidong Gu, Jianing Zeng

**Affiliations:** 1 Bioanalytical Sciences, Research & Development, Bristol-Myers Squibb Co., Route 206 & Province Line Road, Princeton, NJ 08543, USA

**Keywords:** fit-for-purpose, immunoaffinity, LC/MS, protein biomarker, quantitative analysis

Biomarkers are defined as being able to be “objectively measured and evaluated as indicators of normal biological processes, pathogenic processes or pharmacologic responses to therapeutic interventions” [1]. The use of biomarkers represents a competitive advantage in drug discovery and development to improve drug discovery effectiveness, facilitate dose selection, evaluate pharmacodynamic (PD) effect and target engagement, assess safety and select patients for treatment. There are many analytical platforms for biomarker measurements including ligand-binding assays (LBAs), MS, flow cytometry and molecular profiling methodologies such as DNA sequencing and immunohistochemistry. Some of these technologies provide quantitative results while the others are considered semiquantitative or qualitative. For soluble marker measurement, LBAs are broadly applied due to their supreme specificity, sensitivity and high sample throughput. However, this methodology highly relies on the specificity and quality of the reagent (antibody), and in most cases requires a pair of antibodies which sometimes may not be available in the early phase of drug discovery and development. In addition, when measuring a drug target for large molecule therapeutics such as monoclonal antibodies, a sandwich LBA requires two different functioning binding sites on the analyte to measure the drug-free form and three binding sites to measure the drug-bound form. This is a great challenge especially for small protein biomarkers, which may not have multiple epitopes available or where the access to the nearby epitopes may be blocked after being bound to monoclonal antibody therapeutics.

## Hybrid immunoaffinity LC–MS approach for quantitative analysis of protein biomarkers advantages

LC–MS-based assays have been widely used in the quantitation of small-molecule drug candidates, metabolites and biomarkers [2]. With recent advances in instrumentations and sample preparation technologies, LC–MS-based assays have been expanded to the bioanalysis of protein therapeutics and protein biomarkers using SRM detection of one or more signature peptide(s), which is generated from enzymatic or chemical digestion and is specific to the protein of interest [3,4]. The major advantages that the LC–MS assays could offer are fast method development, ease for multiplexing, good selectivity and assay reproducibility. Moreover, the recently developed hybrid immunoaffinity enrichment approach that utilizes specific or nonspecific reagents to pull down and enrich analytes from biological matrix removes the sensitivity obstacle and makes LC–MS a more useful technology in the quantification of low-abundant proteins [5]. The enrichment could be performed either on the protein level or on the digested peptide level using either antiprotein or antipeptide antibodies [6]. The supreme assay specificity is provided by the antibody specificity during immunocapture, chromatographic separation on LC columns and MS selectivity on mass analyzers. Therefore, unlike the ligand-binding assays, there are less requirements on the specificity of the capture antibody for the hybrid immunoaffinity LC–MS/MS assays. For example, in the cases where the capture antibody cannot differentiate the biomarker of interest from a structural similar interference component, LC–MS may provide the solution as long as there is a sequence difference between the two molecules [7]. The hybrid LC–MS assay also provides the advantage of multiplexing such as simultaneously measuring two or more structurally similar biomarkers with only one antibody pull down, followed by MS detection. [8]; quantifying both the therapeutic drug and its target to evaluate target engagement using mixed beads approach [7] as well as monitoring different isoforms of the biomarkers [9]. Most importantly, since only one capture antibody is needed in the hybrid approach, the requirement for multiple antibodies and multiple binding sites in a sandwich LBA for specificity no longer exists, and therefore, the assay development could be accelerated and the protein biomarker can be evaluated as soon as a single antibody is available in the very early stage of drug development.

## Challenges & important considerations

Due to the endogenous nature of the protein biomarkers and lack of authentic reference materials which are identical to the endogenous counterparts, the quantitation of a biomarker is usually considered as ‘relative quantitation’ rather than ‘absolute quantitation’ as the regular pharmacokinetics (PK) assay. A ‘fit-for-purpose’ approach [10] is generally followed to determine the extent of validation required depending on the stage of the drug development and the purpose of the assay. Scientists need to take into considerations all of the factors that could contribute to the assay variations during method development and validation. Regardless of which analytical platform is used, the main analytical challenges include: lack of a suitable calibrator/matrix, specificity and stability, as well as sensitivity. Prior to starting any assay development, two of the most important questions to be answered are: how do we prepare the calibration standard curve? And does the standard represent the endogenous analyte?

Different from small molecules biomarkers, protein biomarkers are not only endogenously produced but also highly heterogeneous with various structures and binding properties. Since it is difficult to obtain a ‘true’ reference standard, in most of cases, the calibrators used in the assay are recombinant proteins which are produced in different cell lines, and could be structurally different depending on the ways they are produced and therefore may not adequately represent the endogenous analytes. Compared with the endogenous, the recombinant protein may be a different isoform, lack proper folding, miss post-translational modifications (if produced in different systems), and may also be a portion of the full-length protein [11]. All of these structural differences may result in variations in protein structures and biological properties, which are critical in the hybrid LC–MS assay development, particularly for the MS detection. For example, if the calibrator and endogenous molecule have different post-translational modifications such as glycosylation, the developed method may only be able to detect the recombinant form but not the endogenous due to the difference in the sequences. Therefore, it is recommended to generate the recombinant calibrator in the cell lines from the same species as the endogenous biomarker, and experiments must be performed to ensure that the developed method is capable of measuring the endogenous protein in the same way as the calibrator, and as early as possible by measuring endogenous analyte in different matrix lots from both healthy and diseased populations. As summarized in a recent publication [10], parallelism is a key parameter that demonstrates the proportionality between the endogenous biomarker and the recombinant calibrator, indicating the similar binding properties for the capture antibody to the endogenous biomarker and calibrator. In addition, since the sample matrix likely contains the biomarker of interest, a surrogate matrix from different species or just a buffer could be used for calibration curve. Under such conditions, scientists also need to demonstrate that the protein biomarker dilutes the same way in the surrogate matrix as in the authentic sample matrix.

For hybrid LC–MS assays, if there is any structural difference between the endogenous analyte and the recombinant standard or subject-to-subject variation (e.g., different variants of antibodies), the binding affinity of the capture antibody to the analyte may be affected, resulting in change of recoveries in individual samples which may not be accurately reflected by simply evaluating standard/quality control performance. Therefore, a consistent recovery in the immunoaffinity enrichment step needs to be demonstrated to show the endogenous biomarker can be enriched consistently among different matrix lots. For example, when measuring a biomarker that is used as an indicator of a particular disease or some clinical end points, it is extremely important to ensure that the immunoaffinity enrichment step could achieve a consistent recovery across different populations. Thus the measured difference between healthy versus diseased population is indeed coming from the progression of the disease instead of assay variation. In another situation, there could be an interfering component that also binds to the capture antibody, either a therapeutic drug or an endogenous protein with similar structure. The assay needs to be optimized to achieve consistent recovery depending on the assay design and capture mechanism in the existence of interfering components [12].

Another important aspect is that the completion of an assay validation is not the end of the story. A proper and careful design of how to implement the assay into sample analysis is also a key factor for success. Scientists need to change their mindset from the traditional PK analysis to the biomarker world and keep in mind what is the goal of the study. For example, if a biomarker is to be used as a pharmacodynamic marker to assess the PD effect after the administration of a therapeutic agent, considering the nature of the fit-for-purpose biomarker assay, it is highly recommended to analyze all samples with both pretreatment and post-treatment time points from the same subject together in one run to eliminate batch-to-batch variability. In addition, the in-study evaluation of the assay performance as well as the evaluation of the endogenous analyte stability is also important since the commercial purchased matrix may not reflect the true incurred samples.

In the end, the measurement of protein biomarker is challenging, and it requires a deep understanding of the biological aspects as well as the purpose of the study. Scientists should use their best scientific judgements to make decisions in regards to study design, assay development as well as the required regulatory rigor in order to implement a right solution to better answer the question. The hybrid immunoaffinity LC–MS approach has shown its value in the quantitation of protein biomarkers to facilitate drug development and has gained wider applications across the industry. However, people should keep in mind that certain barriers still exist such as the requirement of the skill set and specialty of the scientist and the cost and availability of instruments when implementing a validated method or transferring assays across labs. Finally, it is important to understand that the hybrid LC–MS approach and ligand-binding assay have their own advantages and disadvantages, and they can serve as a complementary methodology to each other and each of them could provide unique value to answer specific questions.
